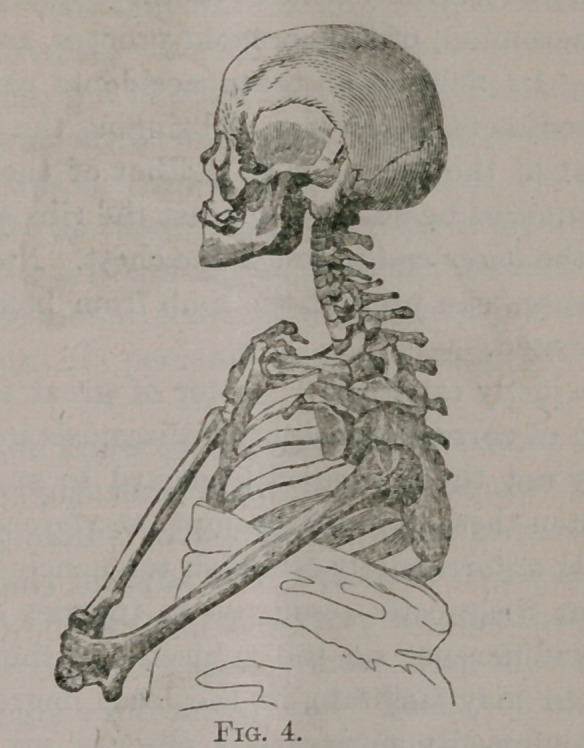# Dugas’ Sign of Dislocations of the Shoulder-Joint

**Published:** 1875-08

**Authors:** W. T. Briggs

**Affiliations:** Professor of the Principles and Practice of Surgery in the Medical Department of the University of Nashville and the Vanderbilt University


### Our Exchanges.

---

DUGAS' PATHOGNOMONIC SIGN OF DISLOCATIONS OF THE SHOULDER-JOINT.—*By W. T. Briggs, M.D., Professor of the Principles and Practice of Surgery in the Medical Department of the University of Nashville and the Vanderbilt University.*—Notwithstanding the great progress in our diagnostic knowledge of injuries about the joints in the last quarter of a century, many errors are still committed, even by able practitioners. The personal experience of every surgeon of large practice will furnish numerous examples of cases in which fractures about the joint have been mistaken for dislocations, and conversely, dislocations for fractures, and simple contusions for either. Now, the signs of each of these injuries are so marked, when the patient is seen early, that even the more inexperienced can scarcely fail to differentiate them; but in a short time so much tenderness and tumefaction supervene, that the signs which were so prominent become completely obliterated, to become prominent again when the tumefaction subsides.

It is during the stage of inflammation and tumefaction that so many errors are made, especially in dislocation of the shoulder-joint; the luxation is left unreduced, and the patient remains a living memorial of the surgeon's ignorance or inattention.

Almost all authors concur in the opinion that it is not always an easy matter to make a correct diagnosis in shoulder-joint dislocations. In 1832, Sir Astley Cooper, in his great work on Dislocations, after discussing the diagnosis of shoulder-joint dislocations, writes: "Yet it would be an act of injustice not to acknowledge that the tumefaction arising from extravasation of blood, and the tension resulting from the inflammation which frequently ensues, will, in the early days of the accident, render it difficult for the best surgeon perfectly to ascertain the exact extent of the injury."

In 1872, Dr. Gross, in his masterly work on Surgery, says: "Although the signs of this dislocation are generally well marked, there are few accidents which are so liable to be mistaken, and no pains should therefore be spared to establish a correct diagnosis."

And Ashurst, in his excellent work on Surgery, says: "Although, by a careful and systematic examination, the true nature of the injury may almost always be eventually determined, the surgeon should hesitate before criticising another practitioner for a mistake that may have been unavoidable under other circumstances."

Such is the opinion of almost every writer on the subject, and if we depend alone on the signs usually mentioned, mistakes will be constantly made. Fortunately, however, we have a sign upon which we can rely implicitly, under all circumstances—one which we have been teaching and practicing, for many years with so much confidence, that we would be willing to base a diagnosis on this one sign alone. We of course refer to Dugas' sign.

In the March number (1856) of the *Southern Medical and Surgical Journal*, we find an article by Prof. L. A. Dugas, on a new principle of diagnosis in shoulder-joint dislocations. In May, 1857, he made a report on the same subject to the American Medical Association. It is astonishing that so clear and lucid an exposition of a principle of diagnosis so important to the surgical world—nay, to the whole medical world—should have received so little attention from surgical writers.

Smith, in his excellent *Surgery*, barely mentions Dugas' paper.

Gross, who is usually so correct in all his writings, in his great work on Surgery, states the principle wrong when he says: "Another sign, although not an infallible one, first pointed out by Dugas, of Georgia, is the inability which the patient experiences in touching the sound shoulder with the hand of the injured limb."

Ashurst, who is always willing to give honor to whom honor is due, does not refer to it in his work. Nor do any of the European writers on Surgery mention it.

Hamilton, in his incomparable work on Fractures and Dislocations, refers very properly to Dugas' sign, and in his *Surgery* gives him full credit for priority, and copies largely from his report to the American Medical Association.

We are satisfied that the profession generally do not appreciate its great value, and even professors of Surgery have often failed to avail themselves of it in their teaching.

Dugas' pathognomonic sign of shoulder-joint dislocations may be stated as follows: If the fingers of the injured limb can be placed by the patient, or by the surgeon, upon the sound shoulder, while the elbow touches the thorax, *there can be no dislocation;* and if this cannot be done, there *must be* a dislocation. In other words, it is *physically impossible* to bring the elbow in contact with the sternum, or front of the thorax, if there be a dislocation; and the inability to do this is *proof positive* of the existence of dislocation, inasmuch as no other injury of the shoulder-joint can induce this inability.

The proposition is so lucidly and powerfully sustained by the author, in his report, that we take the liberty of quoting it in full, with the illustrations:

"In order to make these propositions apparent, I beg leave to present drawings, taken from the skeleton, showing the relative position of the bones in the natural state, and in the several dislolations of the shoulder. The evidence thus obtained in support of my principle would be still stronger if the bones were invested with their normal coverings and attachments.

"Let us then look at Fig. 1, and we may observe, that while the head of the humerus occupies the glenoid cavity, and the fingers rest upon the other shoulder, the elbow and lower end of the humerus lie upon the thorax without difficulty, because of the circumstance that the head of the humerus, when in its natural position, is removed several inches from the ribs. In consequence of the rotundity of the thoracic walls, it is physically impossible that both ends of the humerus should, at the same time, come in

FIG. 1.

contact with the chest. We see, therefore, in Fig. 1, that in the absence of any dislocation, the *upper half* of the bone does not touch the thorax, and that the *lower half* does so without the least difficulty.

"By now referring to Fig. 2, which represents a dislocation into the axilla, we find that, the fingers being placed upon the opposite shoulder, the elbow is forced so far forwards that it *cannot* touch the thorax. In this state of things, the upper end of the humerus alone touches the ribs, while the lower end is proportionately removed from the chest. Any attempt to force the elbow against the thorax must be fruitless, unless at the expense of a disruption of all the soft parts by which the head of the humerus is held down; for, as I have already stated, it is *physically*

*impossible* for both ends of the humerus to touch the thoracic walls at the same time.

FIG. 2.

"We have represented, in Fig. 3, a dislocation forwards, or below the clavicle; and here again we find the *upper end* of the humerus resting upon the ribs—the elbow being consequently removed from the chest. The *upper* half of the humerus touches

FIG. 3.

the thorax, and so long as this is the case, it is physically impossible for the *lower* portion of the humerus also to do it. In dislocations of this kind it is very difficult to carry the fingers upon the opposite shoulder, even though the elbow be allowed to project forward, because of the resistance offered by the strong muscles which pull back the humerus. I have, however, represented the bones of the skeleton in this position, for the purpose of showing the effect, in case it could be assumed, in the living subject.

Fig. 4.

Dislocations of the humerus upon the dorsum of the scapula, although very rare, should still be carefully studied. I have, therefore, represented this accident in Fig. 4, by which it may be seen that the same principles are applicable also to it.

"Here, as well as in the other instances, it is only the *upper end* of the humerus that touches the thorax, and the elbow projects strongly forwards. In this dislocation it might be possible to bring the elbow against the side of the trunk, by carrying the the humerus down parallel with the axis of the body; but any contact of the elbow with the chest is impossible, if the fingers be directed towards, or placed upon the sound shoulder, for then the form of the thorax would offer an insuperable obstacle.

"Having now, I trust, sufficiently demonstrated the truth of the proposition that it is *physically impossible* to bring the elbow against the front of the thorax in dislocations of the shoulder, I

would simply add, that it is equally true that no other injury of the shoulder-joint than a dislocation can induce this physical impossibility. It is obvious that a mere contusion of the soft parts may render motion of the joint so painful as to deter the patient from the effort necessary to carry the fingers upon the other shoulder. But there can be no difficulty on the part of the surgeon in placing the limb in this position, and an anæsthetic might be used, if desirable, so as to render manipulation painless. The same may be said of fractures of the upper end of the humerus, of the acromion, of the coracoid process, and of the neck of the scapula. In neither of these accidents can there be any physical impediment in the way of bringing the elbow in contact with the front of the chest, for in neither of them can the *upper end* of the humerus be so fixed against the ribs as to make it impossible for the *lower end* to touch the chest. Nothing, therefore, but a dislocation, can prevent the limb from being placed in the position indicated.

"If it be justly esteemed a matter of great importance to be in possession of correct principles of diagnosis in occult diseases, it is certainly not the less so with regard to surgical accidents, especially when these demand prompt interference. Our professional records unfortunately establish too conclusively the imperfection of our diagnostic resources in injuries of the joints, to permit any indifference on the subject. If, therefore, the views here presented may facilitate, in the least degree, the detection of injuries confessedly more or less obscure, my object will have been attained."

The principle upon which this important diagnostic sign is based is true. The sign is pathognomonic in every variety of dislocations of the shoulder-joint. It is simply perfect and infallible.

Western surgeons* have done much for the general advancement of surgery. McDowell, Sims, Deaderick and Smythe have made their names immortal by their glorious deeds; but Dugas has conferred more benefit on the profession by pointing out this true sign of dislocated shoulder-joint than either of those distinguished men. It should be known and handed down to all future generations as *Dugas' Sign.*

---

*Dr. Bowling was the first to call attention to the fact that American surgery owes most of its progress and glory, during this century, to those who lived in towns and villages of slave States. He calls the names of his heroes his "string of beads."

*Nashville Journal of Medicine and Surgery.*

## Figures and Tables

**Fig. 1. f1:**
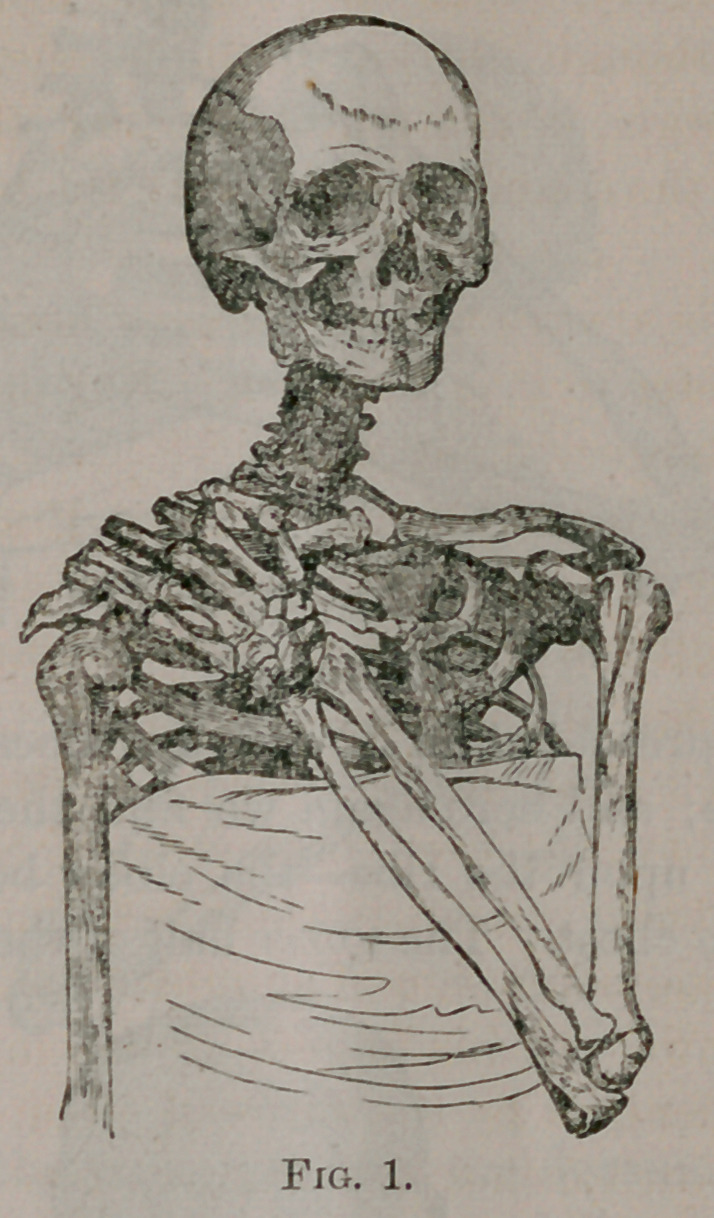


**Fig. 2. f2:**
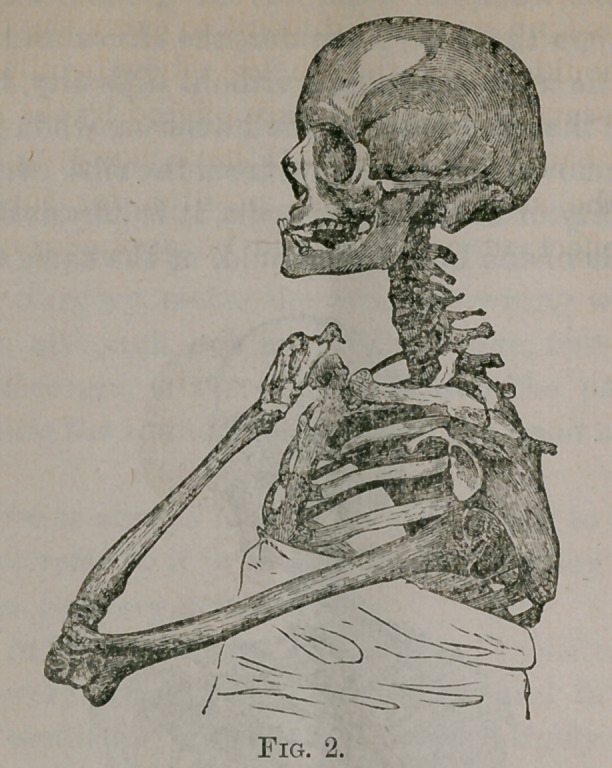


**Fig. 3. f3:**
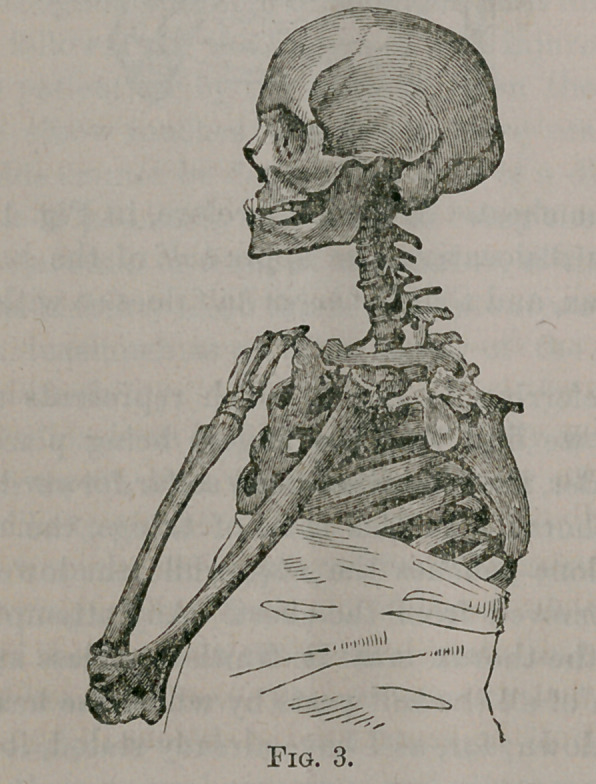


**Fig. 4. f4:**